# Current status of running renewable energy in Bangladesh and future prospect: A global comparison

**DOI:** 10.1016/j.heliyon.2023.e14308

**Published:** 2023-03-15

**Authors:** Md. Abdullah-Al-Mahbub, Abu Reza Md Towfiqul Islam

**Affiliations:** a Dr. Wazed Research and Training Institute, Begum Rokeya University, Rangpur, Bangladesh; b Department of Disaster Management, Begum Rokeya University, Rangpur 5400, Bangladesh

**Keywords:** Renewable energy, Solar energy, Wind energy, Hydropower, Bioenergy, Bangladesh

## Abstract

World's fossil fuels are disappearing rapidly due to multidimensional uses, mainly for electricity generation. Nevertheless, Bangladesh has also a very limited source of natural gas and coal for electricity production. Hence, the major goal of this review is to outlines the present status of installed renewable generations in the country and predict the future prospect. Despite the existence of literature's abundance on Bangladesh's potential for renewable energy (RE), and their prospects, nothing is covered about the phases of renewable energy projects like projects already completed and running, projects implementation ongoing, and projects under planning. Therefore, an endeavor has been made for the first time to expose Bangladesh's three phases of renewable energy, including projects that are currently operational, those that are still being implemented, and projects that are still in the planning stages. Data was collected from Bangladesh Power Development Board (BPDB), Bangladesh Bureau of Statistics (BBS), Infrastructure Development Company Limited (IDCOL), International Renewable Energy Agency (IRENA), Renewable Energy Policy Network for the 21st Century (REN21), International Hydropower Association (IHA), Sustainable and Renewable Energy Development Authority (SREDA), Ministry of Disaster Management and Relief (MODMR), and Ministry of Power, Energy and Mineral Resources (MOPMER). Based on these data, this research suggests that Bangladesh is generating 723.26 Megawatt (MW) electricity from renewable sources including 67.61% from solar, 31.80% from hydro, 0.58% from others including wind, biogas and biomass, where 489 MW electricity makes from over 6 million (63, 25, 278) of installed solar Photovoltaic (PV) systems till mid of April 2021 and a total of 6,408,721 numbers of RE plants are completed and running. Bangladesh is a prospective area for harvesting solar, wind, and bioenergy with limited hydropower, despite the fact that over 42% of rural societies still lack access to electricity. This review will help investors, shareholders, researchers and decision makers of both public and private sector to realize the latest renewable energy situation of Bangladesh, and for future planning and management in a sustainable way.

## Highlights

• Solar, wind, bioenergy and hydropower are the present renewable energy in Bangladesh.
• The Government of Bangladesh has set a goal of creating 2624 MW of renewable energy, of which 723.26 MW are now in production, 519.956 MW are in the implementation phase, and 1328.81 MW are in the planning stage.
• Over 6 million solar PV systems have been installed, producing approximately 489.03 MW of electricity.
• Wind energy would be potential especially in the coastal Bangladesh.
• Bangladesh produces 155.82 million ton of poultry and livestock manure each year which would be potential for bioenergy generation.

## Graphical abstract

**Figure:**
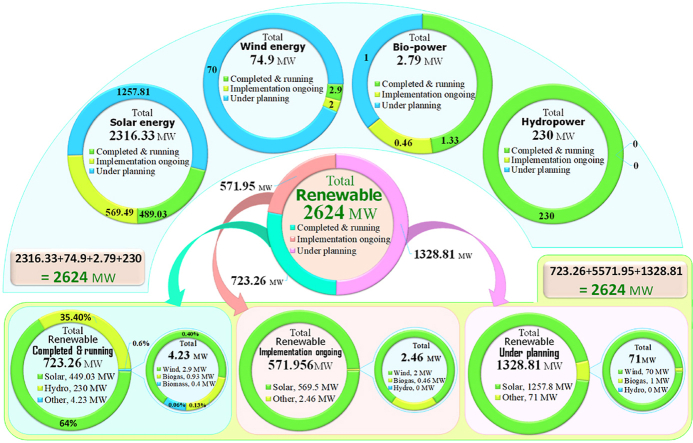

## Nomenclature

**Abbreviations and acronyms in alphabetic order**
BBS: Bangladesh Bureau of Statistics
BCSIR: Bangladesh Council of Scientific and Industrial Research
BPDB: Bangladesh Power Development Board
BREB: Bangladesh Rural Electrification Board, BTS Base Transceiver Station
CSP: Concentrating solar thermal power
EGCB: Electricity Generation Company of Bangladesh Ltd.
EJ: Exajoule, FAME Fatty Acid Methyl Ester, GDP Gross Domestic Product
GHGs: Greenhouse Gases
GIZ: German Agency for International Cooperation
GoB: Government of the People’s Republic of Bangladesh
GW: Gigawatt
GWh: Gigawatt-hour
HFO: Heavy fuel oil
HSD: High Speed Diesel, HVO Hydrogenated Vegetable Oil
IDCOL: Infrastructure Development Company Limited
IHA: International Hydropower Association, IRENA International Renewable Energy Agency
kW: Kilowatt
kWh: Kilowatt-hour
MoDMR: Ministry of Disaster Management and Relief
MoPEMR: Ministry of Power, Energy and Mineral Resources
MW: Megawatt
NEM: Net energy metering / Net metering
RDCD: Rural Development and Cooperative Division
RE: Renewable Energy
REN21: Renewable Energy Policy Network for the 21st Century
SREDA: Sustainable and Renewable Energy Development Authority
TWh: Terawatt-hour

## 1 Introduction

Today energy is synonymous with the development and is the basis as well as a vital ingredient for the development of modern nation and plays an essential role for survival and improvement of human life [1]. Electricity is a must for the world to advance both technologically and sustainably. Its use is expanding globally due to the rate of technological improvement and rising urbanization. Every second electrical instruments are consuming further and further energy due to high-tech progressions, increasing demand, and occasionally, wasteful and consumption of energy inefficient electrical and electronic devices [2,3]. Any nation cannot survive without energy/electricity because it is a controlling engine of technological, social, economic, environmental as well as sustainable development [4]. Energy is necessary for meeting the many needs of humanity, including basic electrification, industrialization, and further progress [5]. All daily activities like charging cell phone, using computer or internet, watching TV or running any other present day's electrical/electronic/IT equipment, are executed by electrical energy. Medical science and all medical checkup and pathological tests cannot imagine without electrical energy. Basically, energy is essential for human satisfaction in every moment and without energy creation of global village is impossible. So, any imbalance relating demand and supply of energy can make vulnerable the country's ability to function, particularly in developing nations [6]. Coal, natural gas, and petroleum are usually specified to fossil fuels [7]. Generally, more or less all nations fulfill their energy demands from conventional energy sources. For example, natural gas is the leading fuel utilized to generate electricity in the Middle East, CIS, North America, and Africa; coal is the principal fuel in Asia; hydropower is the principal source of electricity in South and Central America where over half of power generated from this source; and only Europe follows the fuel diversification strategy for power generation by five diverse fuels like nuclear (the main source), natural gas, coal, renewables and hydro are all in a narrow range of 16–23% [8]. Burning fossil fuels produces roughly three-quarters (75%) of the energy used in the world, which release a huge amount of anthropogenic GHGs mainly carbon, solid substances and other gases as the energy consumption increases; and therefore, it causes pollution, global warming and ultimately climate change [[9], [10], [11], [12], [13]]. It is assumed that the conventional fossil fuel-based electricity generation have added to one-third of worldwide greenhouse gas emissions [14] causing major threats to global present and future environmental safety and security on health and social issues [15]. In 2019, China was the main primary energy consumer in the world, using 141.7 EJ (EJ). The second largest consumer was U.S., using 94.65 EJ followed by India (34.06 EJ), Russia 29.81 EJ, Japan (18.67 EJ), Canada (14.21 EJ), Germany (13.14 EJ), Brazil (12.4 EJ), S. Korea (12.37 EJ) and Iran (12.34 EJ) [16].

Moreover, it has been assessed that world's total fossil fuels will have been totally consumed within a few decades not only that these resources are inadequate and are becoming expensive and scarcer rapidly [17]. In order to ensure energy security, several nations are switching substantially on renewable sources of energy to fulfill their electricity requirements [18]. According to the REN21, global RE generation capacity added up to 3146 GW at the end of 2021 [19].

In Bangladesh, 26 gas fields have been detected [20] till now and the gross gas initially in place (GIIP) is 40.09 Tcf, in which assessed total recoverable gas reserve (2P) is 30.06 Tcf. The cumulative gas production as of December 2020 is 18.24 Tcf, and remaining reserve up to December 2020 is only 11.81 Tcf [21] for next 10–12 years [22], shown in Table S1 and Table S2. Additionally, there are approximately 3139 million tons of high-grade bituminous coal reserves, which would be equivalent to 85 Tcf of gas in five coal fields, namely Jamalganj (1054 MT), Dighipara (600 MT), Phulbari (572 MT), Khalaspir (523 MT), Barapukuria (390 MT) and shown in Fig. S1 [20].

**Fig. 1:**
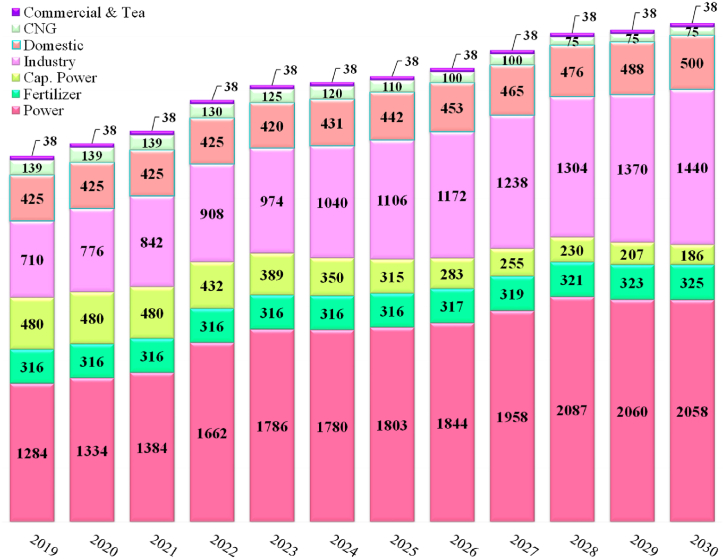
Natural gas demand projection in the country (Unit: mmcfd) [Source: Created by the authors based on [20] data].

**Fig. 2:**
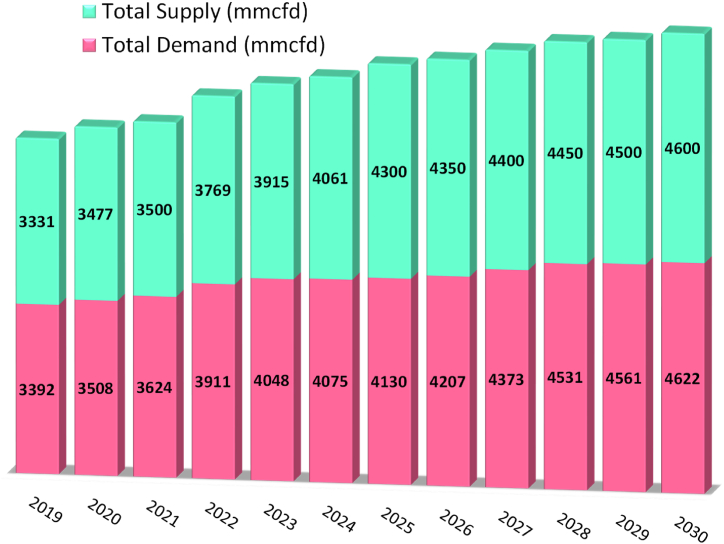
Natural gas supply and demand (Unit: mmcfd) [Source: Created by Authors based on HCU [20] data].

Over the last three decades, the demand of natural gas increased very fast in Bangladesh. Fig. 1 presents the natural gas demand projection in the country [20]. The present natural gas demand is about 3624 MMscfd whereas supply is 3500 MMscfd (Gas + imported LNG) representing a deficiency of about 124 MMscfd and the demand will growth to about 4622 MMscfd by the year 2030, shown in Fig. 2.

Sustainable energy sources are being used by developing countries to meet their energy needs, including Bangladesh, Pakistan, Malaysia, Vietnam, among others [23]. Bangladesh is a favorable location for the increase of the production of RE from a geographical perspective and the geographic position offers the nation a number of advantages that allow it to thrive in the generation of sustainable renewable energy. Bangladesh is a location with boundless potential, natural and human resources. To effectively use its resources to address the problems it is now experiencing, the nation lacks a methodical approach. Bangladesh is still working to better its economic situation despite the fact that the majority of its population live in poverty, and its GDP recently increased by 11.33% from 2020 to 2021 [24].

The production of electricity primarily depends on natural gas, oil, and coal―the reserve of which is limited in Bangladesh [25] as well as emit a lot of greenhouse gases. For this reason, the GoB has recently taken an initiate to enhance environment friendly RE generation capacity along with conventional energy sources which will play an essential role to meet the energy demand in future. However, the majority of people are not concerned about Bangladesh's renewable market or the advantages of renewable energy. This information gap is a significant obstacle to the widespread use of RE.

There have been many studies on Bangladesh's RE status, prospects, and potential, a few of which are [1,3,4,[26], [27], [28]], however none of them have yet revealed the details of running renewable developments i.e., projects completion and running, projects implementation ongoing, and projects under planning stage and their future prospects. They all explored very briefly the present status and future prospects of renewable energy in their articles. Therefore, the main goal of this review is to review and identify the current status of RE progress. The novelty of this study is to recognize the current status of running RE progress in terms of RE plants completion and running, projects implementation ongoing, and projects under planning stage in Bangladesh and to predict their future potential (Table 1).

**Table 1:** Present status of RE capacity from various sources in Bangladesh [Created by the authors based on SREDA data [40]].

RE Source	Project name	Completed and running		Implementation ongoing		Under planning		Rejected from planning phase		Total (scenario before rejection)		Total (present scenario after rejection)	
	All solar projects	S	C (MW)	S	C (MW)	S	C (MW)	S	C (MW)	S	C (MW)	S	C (MW)
Solar	Solar park	5	88.4	10	565.16	19	1257	2	200	36	2110.56	34	1910.56
	Rooftop solar except net metering	117	39.394	2	0.54	2	0.813	1	0.25	122	40.997	121	40.747
	Net metering rooftop solar	1304	23.169	0	0	0	0	0	0	1304	23.169	1304	23.169
	Solar irrigation	2102	44.531	96	3.796	0	0	0	0	2198	48.327	2198	48.327
	Solar mini-grid	27	5.656	0	0	0	0	0	0	27	5.656	27	5.656
	Solar nano-grid	2	0.001	0	0	0	0	0	0	2	0.001	2	0.001
	Solar charging station	14	0.278	0	0	0	0	0	0	14	0.282	14	0.282
	Solar home system	6,023,631	262.75	0	0	0	0	0	0	6,023,631	262.75	6,023,631	262.75
	Solar street light	296,061	16.7	0	0	0	0	0	0	296,061	16.7	296,061	16.7
	Solar powered telecom BTS	1933	8.06	0	0	0	0	0	0	1933	8.06	1933	8.06
	Solar drinking water system	82	0.095	0	0	0	0	0	0	82	0.095	82	0.095
	***Total solar projects***	***6,325,278***	***489.034***	***108***	***569.496***	***21***	***1257.81***	***3***	***200.25***	***6,325,410***	***2516.6***	***6,325,407***	***2316.347***
***Wind***	***All wind projects***	***3***	***2.9***	***1***	***2***	***3***	***70***	***0***	***0***	***7***	***74.9***	***7***	***74.9***
***Biogas***	***Biogas to electricity***	***7***	***0.93***	***2***	***0.46***	***1***	***1***	***0***	***0***	***10***	***2.39***	***10***	***2.39***
	***Biogas plant***	***83,431***	***-***	***-***	***-***	***-***	***-***	***-***	***-***	***83,431***	***-***	***83,431***	***-***
***Biomass***	***Biomass to electricity***	***1***	***0.4***	***0***	***0***	***0***	***0***	***0***	***0***	***1***	***0.4***	***1***	***0.4***
***Hydropower***	***All hydro projects***	***1***	***230***	***0***	***0***	***0***	***0***	***0***	***0***	***1***	***230***	***1***	***230***
**Total renewables**		**6,408,721**	**723.264**	**111**	**571.956**	**25**	**1328.81**	**3**	**200.25**	**6,408,860**	**2824.29**	**6,325,426**	**2624.037**

(S= System; C=Capacity; MW = Megawatt).

The remaining portions of the paper are arranged into eight segments. The methodology is covered in Section 2, which is divided into two units called data source and systematic structure for conducting the review task. The generation of energy (power) in Bangladesh is covered in Section 3. The current status of global renewable energy is described in Section 4. The current status of the various operating RE sources in Bangladesh, which are broken down into solar energy, wind energy, bioenergy (biopower, biofuel, biomass, and biogas), and hydropower, is explained in Section 5. Future prospects of RE in Bangladesh are discussed in Section 6. The policy for renewable energy is presented in Section 7. Discussion is deliberated in Section 8. Finally, the conclusion brings the paper to a close.

## 2 Methodology

For doing in-depth research, pertinent data must be obtained through scholarly articles, yearly reports, or official websites. The most important duty before writing reviews of any topic is choosing relevant keywords. 230 academic literatures are found based on the selected keywords. Only 221 items are gathered from the thousands of articles returned by the keyword search after careful filtering. Additionally, 112 papers are discarded and 109 articles are finalized based on the acceptability and relevance of this review process. In addition, 69 academic journal (67 journals, 2 conference proceedings), and 19 annual reports from different RE organizations, and 21 official website databases from the grey literature are used. The recommended review task is then presented after carefully analyzing 106 articles and documents.

### 2.1 Systematic structure for carrying out the review work

**Fig. 3:**
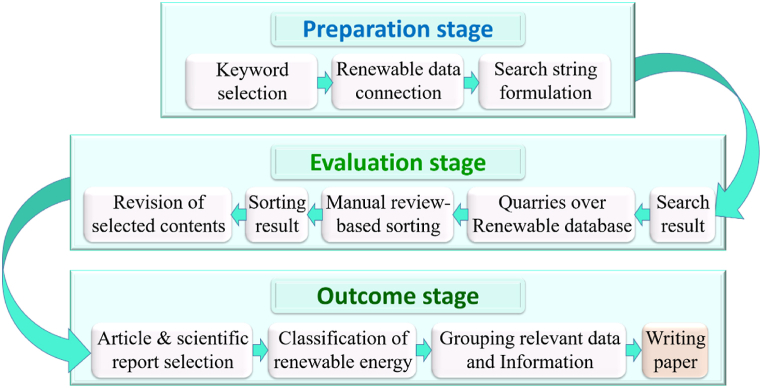
Review methodology.

A well-planned approach becomes the main necessity for conducting systematic research. Preparation stage, evaluation stage, and outcome stage are the three primary divisions of the research approach, as illustrated in Fig. 3. The subsequent subsections give more information about these. Fig. 3 depicts the systematic structure for carrying out the review work.

2.1.1. Preparation stage: The systemic research process begins with the preparation phase. The research's goals are developed during this stage. Then, in order to achieve the goal, a set of key phrases was chosen in relation to the primary goals, and a search was carried out. The primary databases for searching research articles include those from Elsevier, Springer, Sage, IEEE, MDPI, and Taylor & Francis. Government websites pertaining to power and RE are also considered. Energy related Reports from different energy websites are also considered as well.

2.1.2. Evaluation stage: Searches are conducted in the chosen databases using keywords and questions related to the research topic. After doing a search using the specified parameters, the found research papers, review articles, and annual reports are examined. Only excellent research sources are chosen after the screening procedure. Any of these materials that do not adhere to the objectives of the research are rejected after additional consideration.

2.1.3. Outcome stage: Information is taken from the chosen research sources in this phase. The data is divided into a number of essential components. Then, pertinent information/facts are compiled and finally, the writing of the article is completed using this systematic technique.

### 2.2 Data source

The primary focus of this study is on Bangladesh's current situation with regard to renewable energy and its potential to spread over the entire nation. The statistics and information came from official reports that were issued by a number of international and national energy agencies and organizations. The following is a list of the studies referenced for this paper.

a) Statistical Review of World Energy 2020, published by the British Petroleum (BP) [8]
b) Global Status Reports (Renewables 2021, 2016, & 2017) published by the Renewable Energy Policy Network for the 21st Century (REN 21) [19,29,30]
c) Hydrocarbon Unit (HCU), Energy and Mineral Resources Division, Ministry of Power, Energy and Mineral Resources [20,31]
d) World Energy Outlook 2019 by the International Energy Agency (IEA) [32]
e) International Energy Outlook 2019 by the U.S Energy Information Administration [33]
f) Annual Report 2021–2022 of Bangladesh Power Development Board (BPDB) [34]
g) Bangladesh Statistical Pocket Book 2019. Bangladesh Bur. Stat (BBS) [35]
h) Power System Master Plan (PSMP) published by the Gov. of the People's Rep. of Bangladesh [36]
i) Power and Energy Sector Strategy Paper (SSP) by Ministry of Planning, Gov. of the People's Rep. of Bangladesh [37]
j) Global Wind Report by Global Wind Energy Council (GWEC) [38]
k) Hydropower Status Report 2020. Int. Hydropower Assoc [39].
l) Sustainable and Renewable Energy Development Authority (SREDA). Ministry of Power, Mineral and Energy Resources [40]

Therefore, the main goal of this review is to determine the current status of RE resources in Bangladesh and to predict their future potential.

## 3 Electricity (power) generation in Bangladesh

**Fig. 4:**
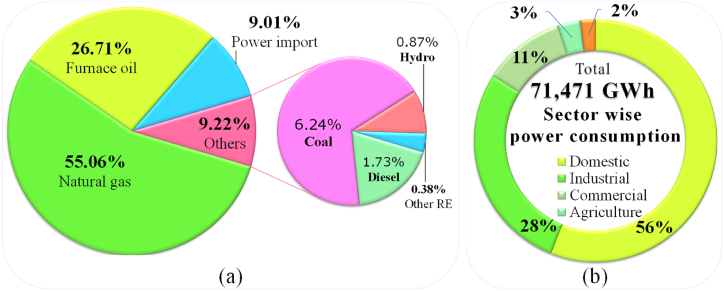
(a) Sources of total net electricity generation in Bangladesh (2021–2022) [20,34], (b) sector wise power consumption pattern in Bangladesh (2021–2022) [34] [Source: Created by Authors based on HCU [20] & BPDB [34] data].

According to BPDB, the current progress of annual energy demand is around 10%, but it will increase in the future due to rising population, their social, economic, technical as well as GDP progressions [34]. In fiscal year 2020–2021, the net electricity production was 80,423 GWh, which was 12.61% advanced than the net production from the prior year of 71,419 GWh. The per capita electricity production in the year 2020–2021 was 560 KWh [35]. In fiscal year 2021–22, the total electricity production was 85,607 GWh, which was 6.45% advanced than previous year's net production of 80,423 GWh. In that year 55.06% (47,136 GWh) electricity was generated from natural gas, 26.71% (22,867 GWh) from furnace oil, 9.01% (7712 GWh) power was imported from India and 9.22% (7892 GWh) was generated from other sources including 1.73% (1483 GWh) from diesel, 6.24% (5342 GWh) from coal, 0.87% (744 GWh) from hydro and 0.38% (323 GWh) from other renewable sources, shown in Fig. 4 (a). In the last year, the sector wise power consumptions were used 57% (40708.83 GWh) in domestic, 28% (19997.32 GWh) in industrial, 10% (7141.9 GWh) in commercial, 2% (1428.38 GWh) in agricultural and 3% (2142.57 GWh) in other sectors respectively, shown in Fig. 4 (b).

**Fig. 5:**
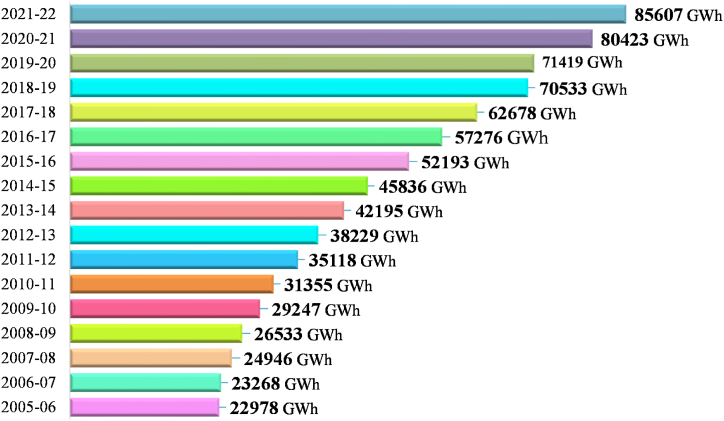
Historical total electricity production in Bangladesh for the fiscal year 2005–2021 [21].

According to the updated PSMP-2016, in 2021, 2030 and 2041, the electricity generation capacity prerequisite will be 21,000 MW, 31,000 MW and 57,000 MW respectively in contrast to the demand of 14,500 MW, 27,400 and 51,000 MW [38,41,42]. The historical total electricity productions in the country are shown in Fig. 5.

Bangladesh, which located in South Asia, is the 8^th^ most populous countries of the world with a population of 168.25 million and has a population density of 1265 people/Km^2^ (3277 people/sq. miles) [43,44]. Over the past ten years, Bangladesh has been suffering from an astounding lack of energy supply in order to fulfill the rising demand [[45], [46], [47]]. Although, in 2019, electricity extended about 93.5% people in the country as a source of lighting up their homes, compared to 90.1% in the previous year [48]; still 79% users experience low voltage supply and 60% users suffer load-shedding. Insufficient energy along with load-shedding and low voltage supply also hinder work activities, reduce exports and sluggish the economic development. Therefore, the nation's energy crisis is a burning issue to fulfill the sustainable future energy demand. So, in order to address the energy crisis, there is no other solution without RE.

In the country's economic history, the GDP growth rate of Bangladesh for 2021 was 6.94%, a 3.49% increase from 2020 [49]. The continuation of the growth of GDP is the precondition of any nation's development and the energy is the main driving force. Basically, sustainable future economic progress ominously depends on the availability of energy or electricity. Therefore, sufficient energy production and proper use of energy is crucial for Sustainable Development Goals.

## 4 Current status of global renewable energy

Energy sources that is really infinite i.e., never run out; that can be easily replenished constantly; that can be generated from natural resources for example sunshine, wind, flowing water, ocean tide, ocean wave, earth's internal heat, biomass and replaceable fuel (plants) is called renewable energy source. These energy sources that can be converted to electricity which is stored and transported to our homes, offices, or industries for use called renewable energy (RE). Alternative energy sources such as RE have play a crucial role to decrease the demand of fossil fuel and could perform an essential role in the energy safety of Bangladesh [50]. RE can be used as fuels for transportation, generation of electricity, and generation of heat [51].

According to Ref. [52], the world's energy consumption is increasing rapidly as compared to population growth. The global commercial progress and rising population will lead about 30% additional energy consumption as the global community has committed to achieve the SDG goals by 2030 and RE can plays an essential role to face this situation (Division P, 2016). In 2017, renewable energy sources generated about one-fourth of the world's electricity [53].

**Fig. 6:**
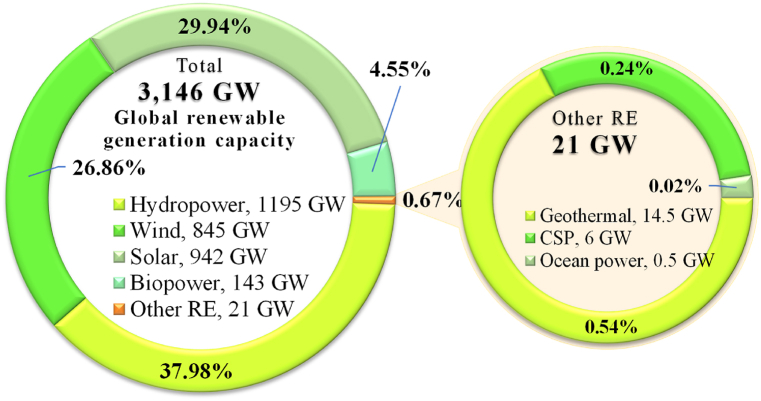
Worldwide energy production capacity by renewable sources in 2021 [Authors creation based on REN21 [54] data].

**Fig. 7:**
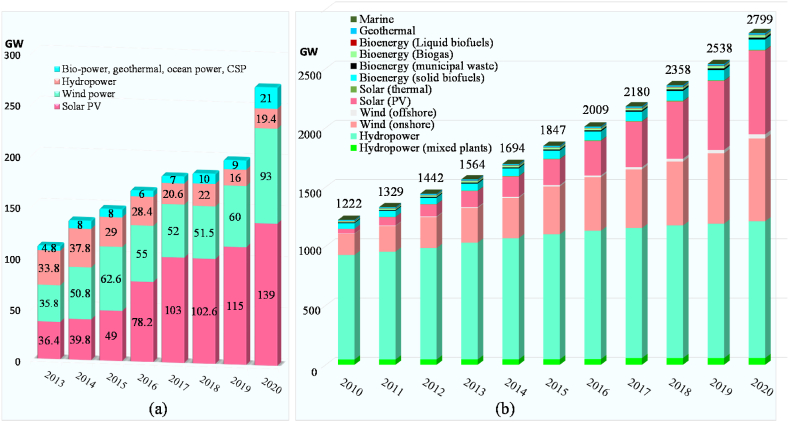
(a) Energy production capacity from renewable sources worldwide, annual new additions (2013–2020) [54]. (b) Worldwide historical renewable production capacity by energy (2010–2020) [Authors creation based on IRENA [55] data].

Fig. 6 shows the world's RE production capacity by energy in 2021. In 2021, the world's total renewable generation capacities come to 3146 GW, which was 306 GW up from the former year's production capacity of 2840 GW (18,41). Hydropower recorded for the highest portion of the world's total RE production of about 37.98% with a capacity of 1195 GW. Wind and solar counted for 26.86% and 29.94% with a capacity of 845 GW and 942 GW. Other renewables counted for 143 GW of biopower, 14.5 GW of geothermal 6 GW of CSP 0.5 GW of ocean power [54]. For the period of the year 2019, more than 272 GW of renewable generation capacity were added where, around +58% of solar photovoltaics (PV) was installed followed by wind power (+29%), hydropower (+8%), bioenergy (+4%), geothermal (+0.5%) and concentrating solar thermal power (CSP) (+0.5%) shown in Fig. 7 (a). It is noted that, solar and wind energy together continued to lead of around +87% of total renewable additions where hydropower expansion was very low. Fig. 7 (b) shows the historical global renewable generation capacities from 2010 to 2020. According to REN21 [54], about 3146 GW RE produced in 2021 including hydropower, wind, solar, bioenergy, geothermal and marine where 1222 GW RE produced in 2010.

## 5 Current status of running renewable energy in Bangladesh

**Fig. 8:**
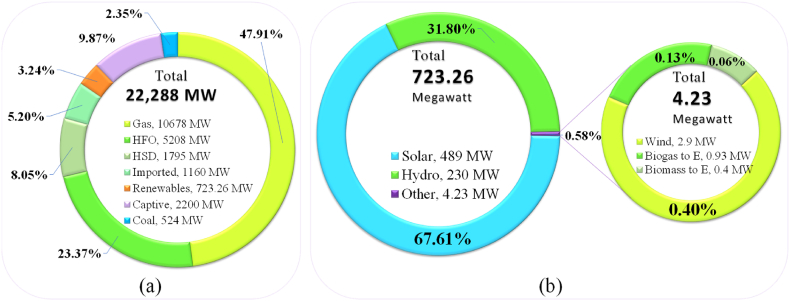
(a) Current electricity generation mix of Bangladesh. (b) Current status of RE production capacity of the country [Source: Created by the authors based on [40]].

As the fossil fuel depleting fast, the GoB has taken several strategies to expand and progress the RE sector along with conventional nonrenewable energy sources. According to Ref. [36], the country's demand of energy can be fulfilled by exploiting RE resources and the renewable generation capacity share will become 5% by 2015, 10% by 2021 and 100% by 2050 respectively [19,36]. The Government has already engaged with several strategies for investment in both public and private division to realize the target [25,56]. The GoB has so far produced 649.95 MW of RE in accordance with the plan, shown in Table 1. Although the GoB has taken a target for generating 1676 MW of solar power by 2021 [19]. Fig. 8 (a) shows the up-to-date electricity generation mix of Bangladesh, and Fig. 8 (b) shows the current status of RE production capacity of the country [40]. Till mid of April 2021, 47.91% electricity was produced from natural gas, 23.37% from HFO, 8.05% from HSD, 5.20% from imported, 3.24% from renewable, 9.87% from captive and 2.35% from coal.

Although, at present, the RE has a tiny amount of share about 3.24% to the net national electricity production. The net electricity generation capacity from renewables reached to 722.592 MW in the mid of April 2021, in which 67.61% (489 MW) from solar, 31.80% (230 MW) from hydro, 0.58% (4.23 MW) from others together with 2.9 MW (0.40%) from wind, 0.93 MW (0.13%) from biogas and 0.4 MW (0.06%) from biomass.

**Fig. 9:**
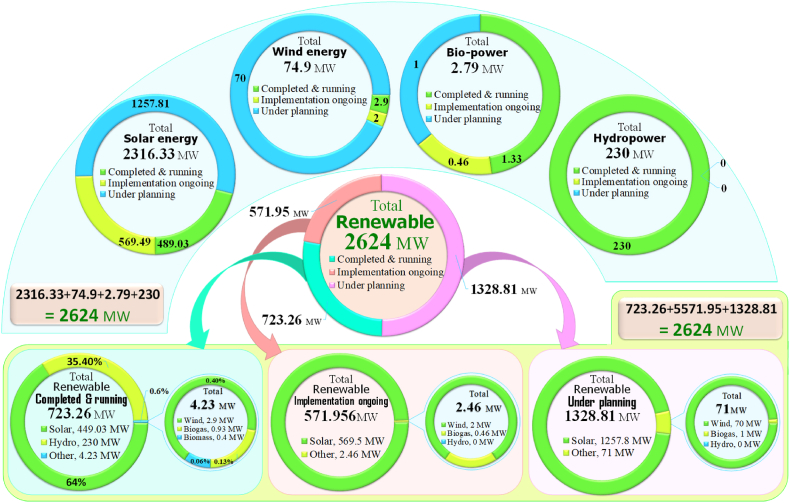
Current status of renewable generation capacity of Bangladesh. [Source: Created by the authors].

The present status of installed renewables capacity in Bangladesh is shown in Fig. 9 & Table 1. The renewable plans in the country is divided into three stages named: (i) RE projects that are already completed and running, (ii) projects that are implementation ongoing, and (iii) projects that are under planning stages. About 723.268 MW of renewable power with a number of 64, 08, 721 renewable projects are completed and running phase; about 571.956 MW with a number of 111 renewable projects are in implementation ongoing phase; around 1328.81 MW with a number of 25 of renewable projects are under planning phase and around 200.25 MW with a number of 3 projects are excluded from planning stage. After rejection, the continuing total numbers of renewable projects (with i, ii, & iii) are 60, 88, 57 with a capacity of 2624.037 MW.

**Fig. 10:**
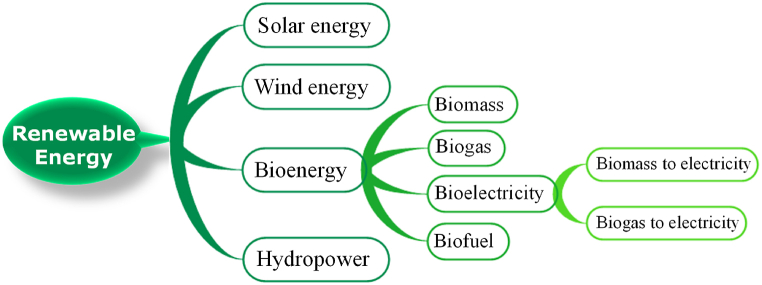
Outline of current RE resources in Bangladesh. [Source: Created by the authors].

The current prominent RE resources of Bangladesh are solar, wind, hydro, biogas and biomass energy, shown in Fig. 10.

### 5.1 Solar energy

Solar energy is a very clean, green and ecofriendly, of all the other renewables and is a giant source for resolving electricity crisis in Bangladesh. The almighty creator creates the sun as a source of all energy, from the agent of photosynthesis to the generation of PV electricity. The sun produces huge energy in every moment on the earth's surface to meet full-year ongoing energy needs [57,58]. Roughly 99.99% energy originates from the sun where earth delivers only 0.01% [59]. It is expected that the sun might release energy of approximately 450 EJ that is comparable to 7500 times more of the global current demand of energy [60].

In the energy sector, solar photovoltaics (PV) is regarded as a standard choice. More and more nations are deploying PV systems to produce more than 20% of their nation's electricity [61]. Brazil, Algeria, Egypt, Mexico, Turkey, Pakistan, and the Netherlands are just a few of the countries that have demonstrated a strong attention in a momentous switch to solar energy over the past three years [55].

#### 5.1.1 Global solar energy scenario

**Fig. 11:**
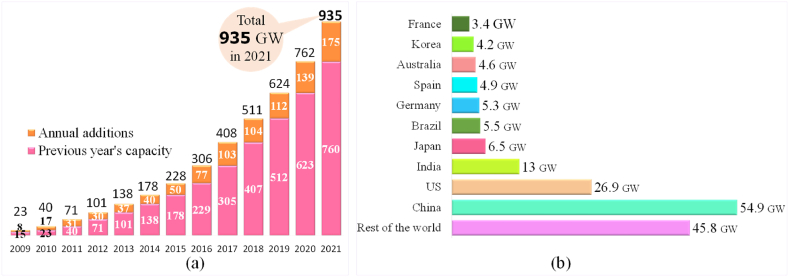
Solar PV global capacity and top 10 countries (a) Solar PV global capacity and annual additions (2009–2021) [19]. (b) Global solar PV capacity additions in 2021, top 10 countries [19].

One of the most affordable and realistic alternative options for generating electricity is solar energy source than other conventional renewables and the demand of solar PV is gradually growing. Fig. 11 (a) shows historical global capacity with annual additions of solar PV from 2009 to 2021 and Fig. 11 (b) shows the top 10 countries of solar energy capacity additions in 2021. In 2021, the global solar PV market expanded about 25% (175 GW) and the volume of total on-grid and off-grid solar PV was 935 GW whereas it was only 23 GW in 2009, just 12 years before. China added the highest capacity of about 54.9 GW (31%), whereas US added 26.9 GW (15%) the second highest capacity of solar energy. India added about 13 GW (7%) the third in globally and the second highest in Asia followed by Japan 6.5 MW (4%), Brazil 5.5 GW (3%), Germany 5.3 GW (3%), Spain 4.9 GW (3%), Australia 4.6 GW (3%), Korea 4.2 GW (2%) and France 3.4 GW (3%). The rest of the countries added about 45.8 GW (20%).

#### 5.1.2 Current status of solar energy in Bangladesh

**Fig. 12:**
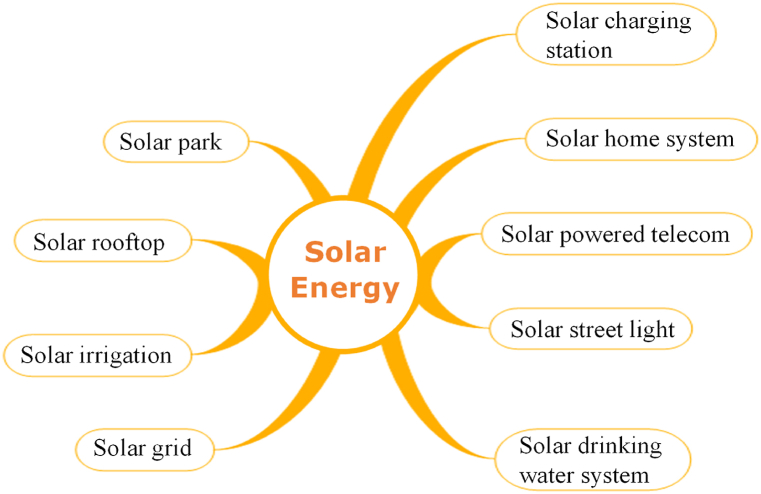
Diversity of solar energy practice in Bangladesh. [Source: Created by the authors].

Solar energy is practiced by diverse arrangements in Bangladesh termed, solar park, solar rooftop, solar irrigation, solar grid (mini-grid and nano-grid), solar charging station, solar powered telecom BTS, solar home system and solar street light [51]. Fig. 12 gives a brief overview of Bangladesh's various solar energy practices.

Although, more than 42% of countryside societies still suffer without electricity access [44]. Bangladesh is generating 723.26 MW electricity from renewable sources where about 489.03 MW electricity makes from over 6 million (63, 27, 278) of installed solar PV systems till mid of April 2021 (Fig. 9 & Table 1). About 569.49 MW with a number of 108 of solar project systems are under implementation ongoing stage; around 1257.813 MW with a number of 21 of solar project systems are under planning stage and around 200.25 MW with a number of 3 systems are rejected from planning stage (Fig. 9 & Table 1). After rejection, the ongoing total numbers of solar systems (including i, ii, & iii) are 63, 25, 407 with a capacity of 2316.347 MW.

Around 125 stakeholders' company of RE in Bangladesh including World Bank, along with other public and private sector like MoDMR, BREB, BPDB, IDCOL, EGCB, RDCD etc. are working with the government and creates one of the world's largest national solar projects. When it came to receiving 8% of its power from off-grid solar energy systems in 2017, Bangladesh was ranked second in the world, after only Nepal [19]. Fig. S2 displays the historical deployed solar power capacity and electricity production in Bangladesh.

### 5.2 Wind energy

**Fig. 13:**
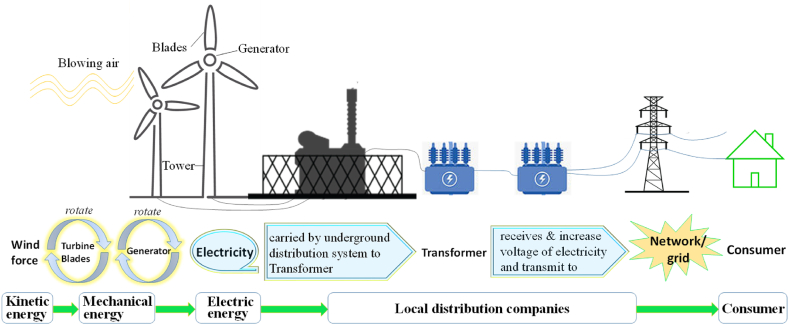
Wind power system, from generation to consumption. [Source: Created by the authors].

Wind energy means making electricity from blowing air which is one of the eco-friendliest RE source. The wind blows against the turbine blades, making them turn. The turbine rotates the generator that makes electricity, shown in Fig. 13. The best locations for installing wind farms are in coastal areas, hill tops, gaps in mountains and other places where the wind speed is favorable. Smaller off-grid wind turbines can be used to power a house or a school, whereas huge wind farms are generally connected to national grid electricity.

Wind power are now extensively used in Americas (USA, Canada, Mexico, Brazil, Chile, Argentina); Africa-Middle East (Egypt, Kenya, S. Africa); Asia-Pacific (Australia, China, Pakistan, India, Japan, Vietnam, South Korea, Thailand, Philippine), Europe (France, Germany, Sweden, UK, Turkey) etc. [38].

#### 5.2.1 Global wind energy scenario

**Fig. 14:**
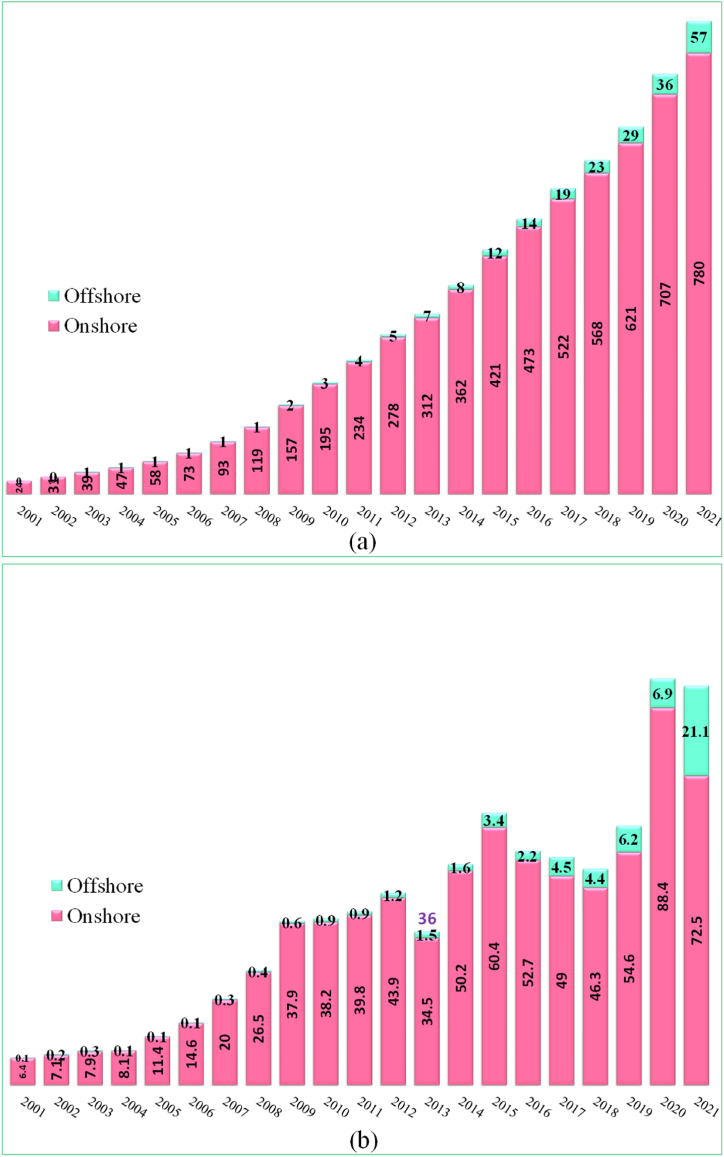
(a) Historic development of total installations of global wind power (2001–2021). (b) Historic development of new installations of global wind power (2001–2021) [Source: Created by the authors based on [38] data].

**Fig. 15:**
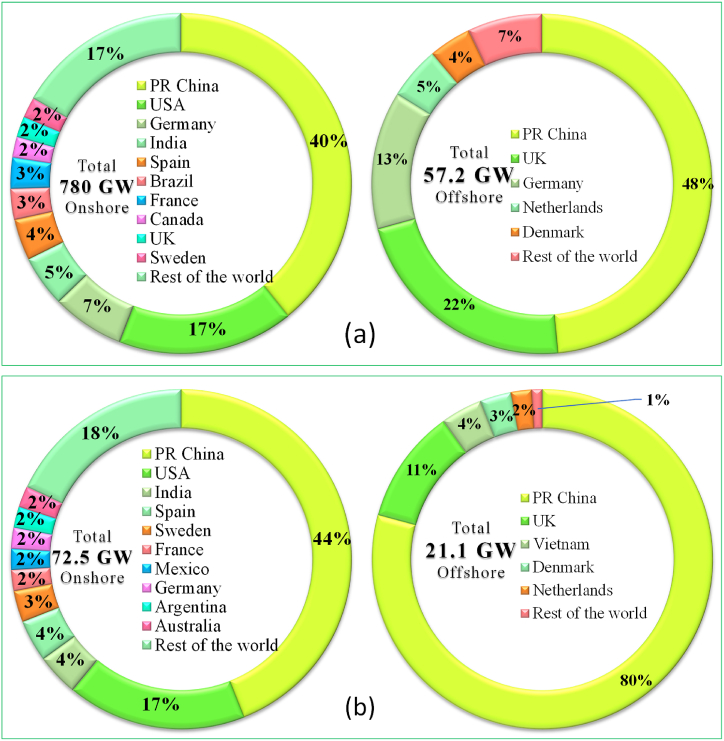
(a) Top 10 countries of global total wind power installation in 2021 (onshore and offshore), (b) Top 10 countries of global annual new additions (onshore and offshore) [Source: Created by the authors based on [38] data].

Fig. 14 (a) shows, the historic development (2001-2021) of total installations of global wind power, and Fig. 14 (b) shows the historic development (2001-2021) of new installations of global wind power. In 2021, global wind energy generated a total of 837 GW (more than 780 GW from onshore and 57 GW from offshore), a progress of 12% compared to 2020, with around 93.6 GW (72.5 GW from onshore and the rest from offshore) of new capacity added to the global grids. With growth in 2021 only 1.8% behind the record year of 2020, the wind sector has experienced its second-best year ever. Despite the COVID-19 pandemic continuing into its second year, about 94 GW of capacity was added [38]. This demonstrates the extraordinary resiliency and rising trend of the global wind sector. Fig. 15 (a) displays the Top 10 countries of global total wind power installation in 2021 (onshore and offshore), and Fig. 15 (b) shows the top 10 countries of global annual new additions (onshore and offshore) in 2021. China and USA together made up 57% onshore total and China and UK made up for 70% offshore total global installation, where as China and USA together made up 61% onshore and China and UK made up 91% of the offshore global new installation [38].

#### 5.2.2 Present status of wind energy in Bangladesh

As Bangladesh is placed in the tropical region, sufficient wind flows all over the year, especially in the southern portion of the country where strong trade wind blows during summer [62]. The mean wind speed in some remarkable locations of Bangladesh is shown in Table S3 [63]. Although, all the areas are not potential for harnessing wind power, the potential locations for wind farms are in coastal zones, offshore islands, at hill tops, riversides and other locations where wind speed is favorable. The coastline of Bangladesh is about 710 km long and extends over 47,201 sq. km area of which about 37000 km sq. is within 50 m depth zone [64,65]. The longest sea beach in the world is situated at the Cox's Bazaar coastline in the Bay of Bengal [66]. In addition, numerous small-sized islands are also situated in the Bay of Bengal. All these areas are potential for harnessing wind energy.

In Bangladesh, 3 wind farms with a combined capacity of 2.9 MW have already deployed and running at Sonagazi (Feni), Kutubdia (Cox's Bazar); another plant with a capacity of 2 MW is under implementation ongoing phase at Sirajganj sadar; and other 3 plants with a capacity of 70 MW are under planning stages at Kalapara (Patuakhali), Maheshkhali (Cox's Bazar) and Chakaria (Cox's Bazar), shown in Fig. S3. Further, the GoB declared plans for 150 MW of new wind plants in the locations of Chandpur, Dakop upazila of Khulna and Inani Sea Beach of Cox's Bazar, each of which a capacity of 50 MW [67,68].

### 5.3 Bioenergy

Energy generated from biological resources or biomasses are called bioenergy. Bioenergy can be renewed into thermal energy (combustions of biomass and biogas directly), biopower (directly by burning of biomass, or altering it into a gaseous fuel or oil, to generate electricity), and biofuels (for transportation).

#### 5.3.1 Biopower

##### 5.3.1.1 Global biopower scenario

According to REN21 (2020) [19], in 2019, biopower capacity of the world rose for a total of about 139 GW, a growth of 6% up from 131 GW compared to 2018. In 2019, the total bioelectricity generation increased about 591 TWh, an increase of 9% up from 546 TW-hours (TWh) in 2018. In that year, the top regional producer was Asia, generating 225 TWh (an increase of 17%) where China produced nearly half of this generation. The second regional producer was EU, generating 200 TWh (an increase of 5%). The North America dropped a little (down 2%) to 76 TWh.

##### 5.3.1.2 Biogas to biopower scenario in Bangladesh

**Fig. 16:**
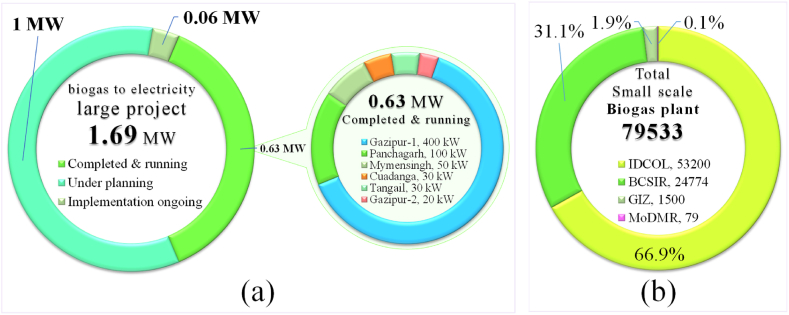
(a) Biogas to electricity large project scenario in Bangladesh. (b) Biogas small project scenario in Bangladesh. [Source: Created by the authors based on SREDA [34] & IDCOL [69] data].

Fig. 16 (a) shows biogas to electricity large project scenario in Bangladesh and Fig. 16 (b) shows biogas small projects scenario in Bangladesh [34,69]. In Bangladesh, primarily six large biogas project systems (capacity 0.63 MW) are in running, one large system (0.06 MW) is in implementation ongoing phase, and another 1 MW grid connected power plant is under planning. There is another biomass based large project system is planning in Thakurgaon, with a capacity of 1 MW.

The installation of small biogas plant has started since the late 1990s, and nearly 80,000 biogas plants have been established and over 60,000 have been under process (Division, 2016). Several organizations like IDCOL, GIZ, BCSIR, MoDMR etc. have been implementing bioenergy project in Bangladesh, where IDCOL has funded building of more than 57,029 biogas plants with daily generation capacity of each plant of about 1.2 m^3^-25.0 m^3^ and stood top position, whereas BCSCIR installed about 24,774, GIG 1500 and MoDMR 128 biogas plant. According to IDCOL, every year these biogas plants protect 51,000 tons of firewood, reduce 45,000 tons of chemical fertilizer by producing 316,000 tons of organic fertilizer and reduce 204,000ton CO_2_ emission per year [69].

#### 5.3.2 Biofuel

The fuel, either gaseous, liquid, or solid, generated directly or indirectly by alteration of biomass is called biofuel for example bioethanol produced from sugar cane or corn, charcoal produced from woodchips, and biogas produced from anaerobic breakdown of wastes [70,71]. Biofuels can also be produced from a diversity of plants like Jamal gota (*Jatropha curcas*), Verenda (*Ricinus communis*), sunflower, sugarcane, mustard, corn, rapeseed, soybean, canola algae, etc. [72].

##### 5.3.2.1 Global biofuel energy scenario

Table S4 shows the global ethanol, Biodiesel FAME and Biodiesel HVO production of top 15 countries [19]. In 2019, the world's ethanol production improved to 113.3 billion litres, above from 112 billion litres in 2018. The top producer was the US, produced about 56%; the second producer was Brazil, produced about 31% of world's production, followed by China, India, Canada, Thailand etc. In 2019, global Biodiesel (FAME) generation increased to 48.5 billion litres, above from 38 billion litres in 2018. The top producer was Indonesia, produced about 7.9%; the second producer was Brazil, produced about 5.9% of global production, followed by United States, Germany, France, Argentina, Spain etc. whereas the global Biodiesel (HVO) production decreased about 6.9 billion litres, fall from 7.3 billion litres in 2018.

The top producers of global biofuel (both bioethanol and biodiesel) electricity productions were US, produced about 433 TWh electricity; the second producer was Brazil, produced about 276 TWh followed by Germany, UK and Mexico. In 1990, the biofuel electricity productions were only 72 TWh in Brazil, 18 TWh in US, whereas Germany, UK and Mexico's production were zero [73].

In Brazil and USA, a vast amount of land and higher technologies are existing for production of food crops and huge amount of raw materials for bioethanol such as sugarcane and corn [74]. In those countries, crop varieties either food crops or cash crops or fuel crops does not matter for land use purposes due to vast amount of land.

#### 5.3.3 Biomass

Biomass is renewable organic substances that originate from animals and plants [75]. In other words, any organic substance prevailing in the environment, whether of plant or animal source, used instantly as firewood or fuel or changed into other methods earlier incineration is called biomass. It covers all varieties of biological resources from fuel wood to aquatic flora such as wood, forest residues, crops residue, vegetal residue, animal dung, rice husk, dead trees, branches, wood chips, and other marine plants [70].

In Bangladesh, the main biomass fuel that are used mainly by rural, remote and low-income people are wood fuels (mainly dead trees, branches, wood chips), agricultural residues (mainly rice husk) and animal dung (mainly cow dung). It is noted that, the urban as well as high income people are used LP gas (as piped or cylinder) for heating and cooking purposes. Over 94% of the rural Bangladeshi people practices traditional solid biomass for inefficient traditional cooking [31] that produce poisonous carbon oxide and particulate matters due to incomplete combustion which is the principal cause of breathing illness. The main victims are women and kids, since they are more exposed to indoor smoke, are three times more probable to hurt from chronic bronchitis or emphysema, than women who cook with gas or electricity. And the use of coal has doubled the risk of lung disease, mostly among women [76].

#### 5.3.4 Biogas

**Fig. 17:**
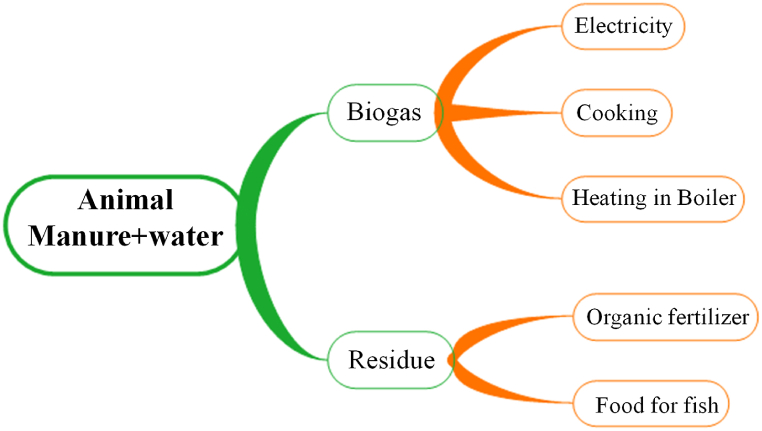
An outline of biogas generation and its uses. [Source: Created by the authors].

Biogas is a mixture of gases in which CH_4_ (40%-70%) and CO_2_ (30%-60%) are dominant, along with H_2_O vapor (1%-5%), N_2_ (0%-5%), and trace amounts of NH_3_, H_2_S and CO [1,3,77,78]. It can be produced from animal's manure (especially cow dung), plant element, or waste (both municipal and agricultural). Biogas plants produce gas along with fertilizer and fish feed as a byproduct, shown in Fig. 17.

In many developing countries, biomass cook stoves and biogas cookers are popular now a days. According to REN21 [19], in Rwanda, about 30,000 cook stoves run by biomass have been given by the government. In South Africa, biomass briquettes and pellets replaced about 33% of the fuels used in clean cook stoves as of 2018. In 2018, about 15.5 billion cubic meters (BCM) of biogas were generated with the aim for cooking where China and India produced 13.1 BCM and 1.6 BCM respectively. At the same year, the demand for biogas for cooking increased in Bangladesh, Nepal and Vietnam. During 2014-2018, African countries like Burkina Faso, Kenya, Tanzania, Ethiopia, and Uganda increased almost 40% to around 46 million cubic meters (MCM) and about 70,000 biogas digesters were mounted in East Africa, benefiting 0.5 million people by clean cooking. Though, the demand for biogas for cooking decreased in China and India between 2014 and 2018.

### 5.4 Hydropower

The global electricity grid system depends on hydropower, one of many renewable energy sources [79]. The greatest noteworthy renewable electricity source in the planet is hydropower and perhaps it was the first renewable resource of energy in the globe [80]. Still now it is supplying the largest amount of renewable electricity of the global total since its beginning. Hydropower means making electricity from flowing water [81]. In most cases a dam is built across a large river and the water is simply sent through a hydroelectric power plant which generates electricity and put into the power lines.

#### 5.4.1 Global hydropower scenario

In 2021, the world's total hydropower production capacity recorded to 1150 GW (44.44%), a major share of the world's entire capacity [19]. Although International Hydropower Association published around 1308 GW of the total generation of hydropower in 2019 which is contradictory from REN21 [39]. But both of the organizations publish the same report about the new addition capacity. Around 50 nations built new generation capacity which was added about 15.6 GW in the year 2019 [19,39].

**Fig. 18:**
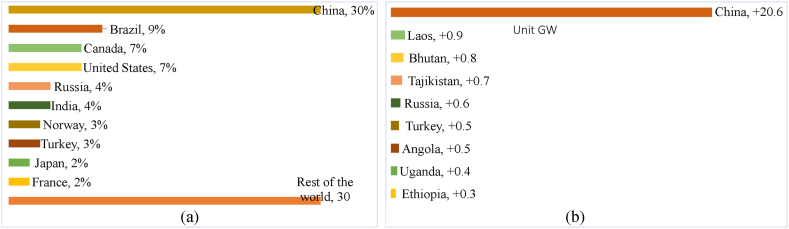
(a) Total capacity of global hydropower generation in 2021, top 10 countries [54]. (b) Total new additions of global hydropower generation in 2021, top 10 countries [54].

Fig. 18 (a) displays the global top 10 countries of the total capacity of global hydropower generation in 2021 and Fig. 18 (b) displays the total new additions of global hydropower generation in 2021. China alone generated about 30%, the highest capacity whereas Brazil generated 9% of hydropower, the second highest capacity. Canada generated about 7% followed by United States 7%, India 4%, Russia 4%, Turkey 3%, Norway 3%, France 2%, and Japan 2%. The rest of the countries of the globe generated about 30%. China alone made up 20.6 GW), the highest capacity additions whereas Laos add 0.9 GW of hydropower. Bhutan added about 0.8 GW followed by Tajikistan 0.7 GW, Russia 0.5 GW, Turkey and Angola 0.5 GW each [19]. Uganda added 0.4 GW, followed by Ethiopia 0.3 GW. Fig. S4 shows the historical global hydropower installed capacity and new additions from 2015 to 2021 [19,30,82].

#### 5.4.2 Present status of hydropower in Bangladesh

Bangladesh has limited potentiality for hydropower due to its nearly flat landscapes with the exception of specific hilly areas of northeastern and southeastern parts [83]. Especially, in southeastern hilly areas, the river Karnafuli, Shangu and Matamuhuri have a lot of tributaries along with several waterfalls having suitable options for installing of micro hydropower. The first private initiatives micro hydropower unit with a capacity of 10 kW has been mounted in Bandarban with a view to supplying electricity to a Buddhist Temple and a number of surrounding 140 households in the village community (55). BPDB has installed a micro-hydro plant having a capacity of 50 kW at Barkal, Rangamati in 2005 and a Mohamaya Hydro Power-cum-Irrigation Project with a capacity of 50 kW-70 kW at Mirersorai, Chittagong [84].

The Karnafuli Hydropower Plant of Kaptai Lake, having a capacity of 230 MW, is the only large hydropower project in Bangladesh. It's No.1 and 2 units (operation started in 1962) and No.3 unit (operation started in 1982) were installed with assistance from the United States. No. 4 and 5 units (operation started in 1987) were installed with support from Japan. With the intention of reinforce the peak demand power supply for, further, No. 6 and 7 units with a capacity of 100 MW were planned as Japanese Yen Loan Projects [20,36]. Besides, at the Sangu and the Matamuhury rivers, 6 km. downstream of existing Karnafuli Hydropower Plant, there are other potential locations of constructing hydropower plant with a capacity of 79 MW [20,65].

## 6 Future prospect of RE in Bangladesh

### 6.1 Future prospect of solar energy in Bangladesh

**Fig. 19:**
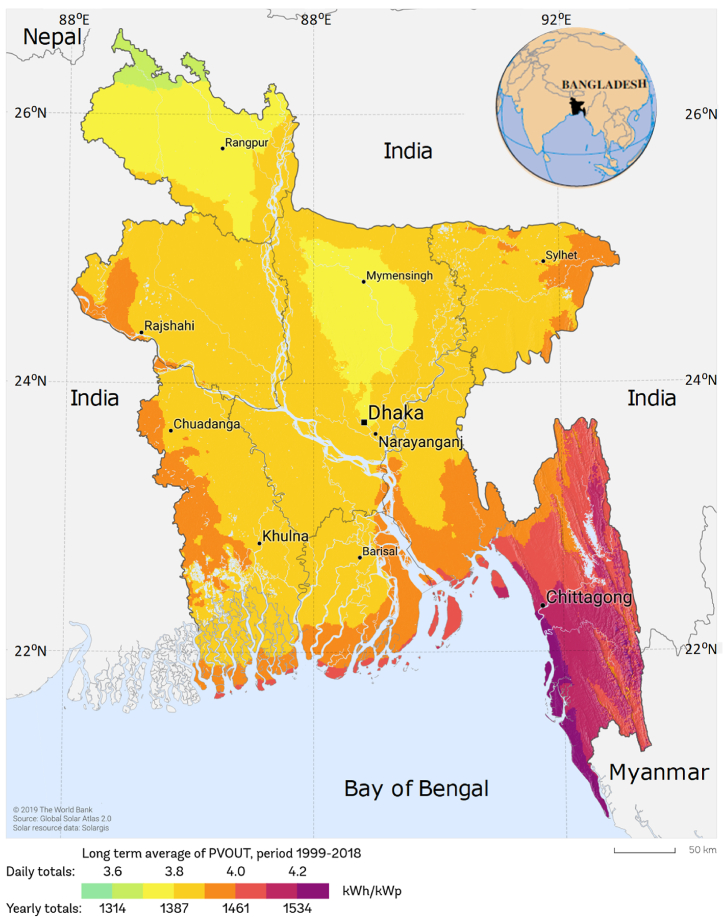
Bangladesh's solar power potential with geographic location [90].

Future infrastructure for generating and distributing electricity must include electric energy storage [85]. Bangladesh is situated in South Asia between 20°34′N to 26°38′N latitude and between 88°01′E to 92°41′E longitude which is a perfect location for solar energy utilization and storage [[86], [87], [88]]. Most of the time of the year sunshine is plentiful for harnessing solar power due to the geographical position of the country [89]. Fig. 19 depicts Bangladesh's solar power potential with geographic location.

The geographical position of Bangladesh is favorable for harnessing solar energy because most of the time of the year sunlight is abundant (Hossain et al., 2017). Extreme solar emission takes place for the duration of March to April and the minimum radiation come about during December and January [2]. With cloud, rain, and fog excluded, Bangladesh has a significant quantity of solar energy available, ranging from 4.0 to 6.5 kWh/m2/day, and sunny daylight hours range from 6 to 9 h/day for about 300 days per year. This indicates that there is enough radiation to meet the need for solar energy requirement from sunlight [10,18]. Bangladesh has a very bright future for solar energy since the GoB has already started implementing various solar projects to provide electricity [91].

### 6.2 Future prospect of wind energy in Bangladesh

The USAID Bangladesh and the NREL (National Renewable Energy Laboratory) partnered with the GoB have develop a national wind resource assessment in 2018, shown in Fig. S5 [65]. According to this assessment, the coastal areas of Chattogram, Barishal and Khulna divisions have wind speed of over 6 m/s at 120-m height, enough for producing wind electricity [92]. The onshore sites are restricted mostly in the southern part of the country that spread about more than 3200 sq. km. and offshore sites are spread within 50–120 km. of the coast. There are at least 150 GW wind power can be generated including 16 GW from onshore and 134 GW from offshore [93]. The map (Fig. S5) also highlights the large area for offshore wind and relatively small area for onshore wind potential.

### 6.3 Future prospect of bioenergy in Bangladesh

#### 6.3.1 Future prospect of biomass to biopower in Bangladesh

**Fig. 20:**
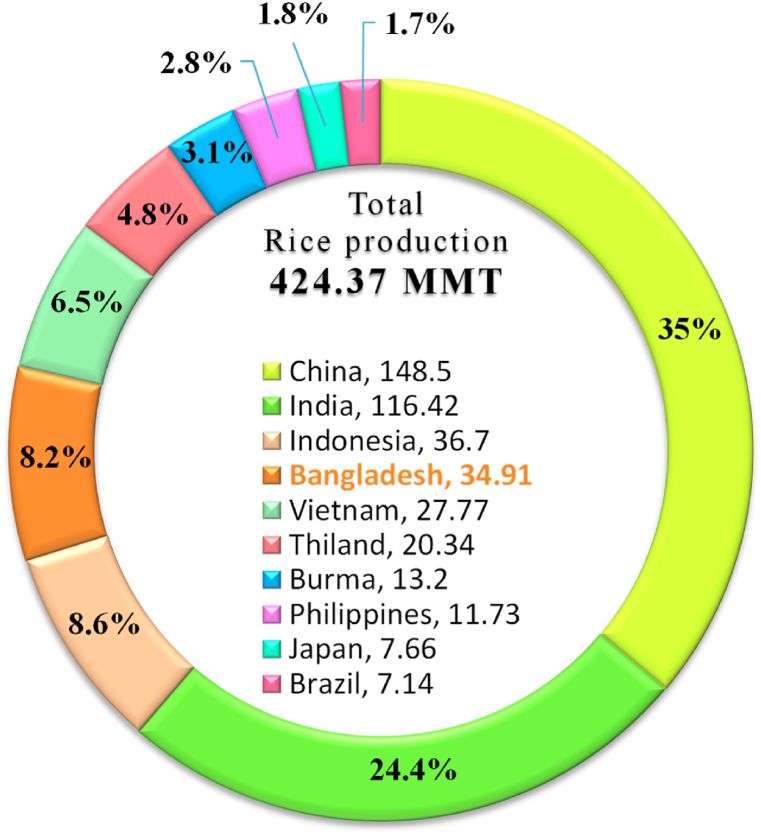
Global rice husk production in the year 2018–2019 [Authors creation based on [95]].

The economy of Bangladesh primarily based on agriculture where rice is one of the chief crops and rice husk is plentiful in the country [94]. Bangladesh, an agricultural country, produces about 97.24 million tons of rice husk and holds the fourth position in global paddy rice production in the year of 2018–2019 whereas global rice husk production was 424.36 MT, shown in Fig. 20 [95]. These rice husk can be used to generate electricity by the conversion from rice husk to producer gas, then producer gas to mechanical energy to run generator for electricity generation. Furthermore, other valuable chemicals such as silica and calcium carbonate (for cement production) can also be produced from the byproducts of this process. In Bangladesh, primarily one biomass to electricity large project system is in running with a capacity of 0.4 MW since December 2015, in Thakurgaon district and the project is financed by IDCOL.

#### 6.3.2 Future prospect of biofuel energy in Bangladesh

In contrast, Bangladesh has higher population and cost with limited land and technology for bioethanol production. It is noted that, density of population of Bangladesh is 3 times higher than nearby India and 36 times greater than United Sates [96]. Therefore, traditional bioethanol/biodiesel appears not feasible for the country. That's why, food production should prioritize than bioethanol/biodiesel production. According to PSMP (Division, 2016), it appears that if Bangladesh demands to follow the biofuel potential, algae technology for biodiesel would be the alternative. More comprehensive site survey is needed about this concern.

#### 6.3.3 Future prospect of biomass in Bangladesh

Biomass, mainly trees (dead leaves, branches and wood chips), has been used as a traditional fuel source from the very beginning of civilization and served as a primary energy source before discovery of fossil fuel. Biomass is still the leading energy source basically for the rural, remote and low-income people of Bangladesh. Although burning of biomass are not ecofriendly, but there is no option without it till they become high income people. But, if the rural communities use biogas, it will improve combustion competence, reduce the poisonous substances and save time/money for collecting/purchasing solid biomass. In addition, if a biogas farm installs a digester, they can generate biogas for their self-consumption, and can sell the remaining gas to neighbor families. Moreover, cooking oil can also be produced from rice husk and cow dung can be used widely as a fertilizer/raw material of biogas.

#### 6.3.4 Future prospect of biogas in Bangladesh

**Fig. 21:**
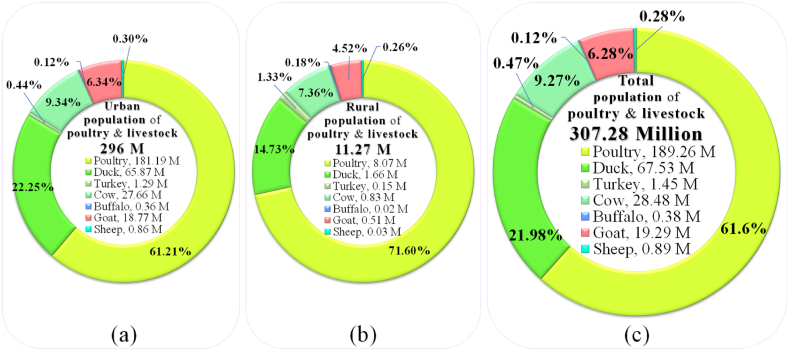
(a) Urban population of poultry and livestock, (b) rural population of poultry and livestock, and (c) total population of poultry and livestock in Bangladesh. [Source: Created by the authors based on MoP [97] data].

Fig. 21 displays the total population of poultry and livestock of Bangladesh. There are roughly 307.28 million of poultry and livestock populations, where 296 million are rearing in rural areas and 11.27 million are in urban areas. Among them, 189.26 million are poultry populations, 67.53 million are duck, 1.45 million are turkey, 28.49 million are cow, 0.38 million are buffalo, 19.29 million are goat and 0.89 million are sheep populations [97]. Every year the country produces 155.8 million ton (155, 820, 000 ton) of poultry and livestock dung [98] and major part of it change into unexploited and polluting environment [99].

The ideal temperature for anaerobic fermentation for biogas generation is around 25 °C – 45 °C with average of 35 °C. Bangladesh's average temperature ranges from 21.2 °C to 30.4 °C, indicate the highest prospective for biogas/bioenergy generation in the country and more than 70% of entire principal energy is provided by biomass mostly by wood and cultivated waste [100].

### 6.4 Future prospect of hydropower in Bangladesh

No recent studies on hydropower potential sites in Bangladesh have been found. Although BWDB and BPDB [101,102] implemented a collaborative study on micro-hydro power in Bangladesh and LGED [103] also compiled a list of suitable sites, shown in Table 2 and Table 3.

**Table 2:** Potential small hydro sites identified by BPDB and BWDB [71,88,101,102].

District	River/Stream/Chara	Potential capacity of electricity (kW)
Chittagong	Foy's Lake	4
	Choto Kumira	15
	Hinguli Chara	12
	Sealock	81
	Lungi Chara	10
	Budia Chara	10
Sylhet	Nikhari Chara	26
	Madhab Chara	78
	Ranga pani gung	616
Jamalpur	Bhugai Kongsa	69kw for 10 months & 48 kw for 2 months
	Marisi at Dukabad near Jhinaigati Thana Head Quarter	35Kw for 10 months & 20 kw for 2 months
Dinajpur	Dahuk at Burabari	24
	Chawai	32
	Talam	24
	Pathraj at Fulbari	32
	Tangon	48
	Punarbhaba at Singraban	11
Rangpur	Buri Khora Chikli at Nizbari	32
	Fulkumar at Raiganj Bazar	48

**Table 3:** Potential Locations for Micro Hydropower in Hill districts [101] and Chittagong Hill Tracts [103].

Location	Potential capacity of electrical energy (kW)
Nunchari Tholi Khal, Khagrachari	5
Sealock Khal, Bandarban	30
Taracha Khal, Bandarban	20
Rowangchari Khal, Bandarban	10
Hnara Khal in Kamal Chari, Rangamati	10
Hnara Khal in, Hang Khrue Chara M ukh, Rangamati	30
Monjaipara micro hydropower Unit	10
Sailopropat, Banderban	5
Madhobkundu, Moulvibazar	15

The neighboring countries of Bangladesh, like Nepal, Bhutan, Myanmar and India, have huge hydropower prospective in near future. Although, Bangladesh has a limited potential for large hydropower plant, the cross-border power exchange could be the best option. Bangladesh can exploit regional hydropower prospective outside its border. According to PSMP, if cross-border imported hydropower is counted as hydropower, Bangladesh can import about 5000 MW hydropower mostly from neighboring countries by 2041 [35].

## 7 Renewable energy policy

By 2041, Bangladesh hopes to be a developed nation and is on track to become a middle-income country. Forecasts prior to the COVID-19 pandemic indicated rising national electricity demand as a result of expanding the economy and population. Bangladesh may diversify its energy mix, lower the risks posed by the erratic prices of fossil fuels, and lessen the detrimental environmental effects of thermal power generation by switching to renewable energy. The Bangladeshi government recognizes the significance of energy as a crucial precondition for reducing poverty and ensuring socioeconomic growth. Over the past few decades, the GoB has approved a number of policies and legal frameworks [104] in harmony with the vision of Article 16 of “The Constitution of the People's Republic of Bangladesh,” which is to eliminate disparities in the living conditions of living between rural and urban areas through electrification and expansion, including:

The principal piece of legislation for environmental preservation is the Bangladesh Environment Conservation Act, 1995 [105], which took effect in place of the previous environment pollution control ordinance of 1992. Through the use of renewable energy, this act placed an emphasis on the generation of clean power. The 1996 National Energy Policy (NEP) assures the best possible growth of renewable energy sources like solar energy, biogas, and biomass fuels. NEP has also suggested the creation of a Renewable Energy Development Agency in Bangladesh to hasten the development of renewable energy infrastructure (REDA). In order to put the Environment Conservation Act, 1995 into effect, the Environment Conservation Rules, 1997 (Amended 2002) propose a set of comprehensive regulations. This legal document also specified a list of projects that fell into the categories of Green, Orange-A, Orange-B, and Red in order to maintain environmental cleanliness. It also suggested adopting these projects by using renewable energy technologies to meet ambient standards for air, noise, and water pollution. The 2002 RE Policy of Bangladesh outlines the duties and funding of organizations that promote RE and affirms the imposition of oil taxes. Additionally, it will guarantee financial incentives for international investors operating in the renewable energy sector by remitting maximum 50% of the salaries of foreign nationals working in Bangladesh. The Bangladesh Energy Regulatory Commission Act, 2003 [106] authorized the energy price for a corporation if their capacity for generating RE is 5 MW or more in compliance with the Power Division of the Ministry of Power, Electricity, and Mineral Resources. In order to promote sustainable energy, the Renewable Energy Policy of Bangladesh, 2008 established Sustainable Energy Development Agency under the Companies Act, 1994. According to this policy, the government may start initiatives for the construction of infrastructure for renewable energy. Bangladesh's 2009 Renewable Energy Policy set a goal of producing 5% of its total consumable energy from renewable sources by 2015 and 10% by 2020.

To achieve its desired GDP growth rate of 7.3% in the Sixth Five-Year Plan, the Power Sector Master Plan (PSMP), 2010 [36], blends RE with traditional energy to transport power to the state energy infrastructure. The GDP of Bangladesh increased by 6.3% between 2011 and 2015. The SREDA is established by the Sustainable and Renewable Energy Development Authority Act, 2012 (Act No. 48 of 2012) [107], which establishes SREDA as the main regulatory body in charge of coordinating all initiatives and projects involving renewable and sustainable energy sources. Additionally, the act confirms Bangladesh's 2009 Renewable Energy Policy's aim for the generation of renewable energy. The Energy Efficiency and Conservation Rules, adopted in March 2015, set a goal for Bangladesh's generation of renewable energy of 15% by 2021 and 20% by 2030. The 2016 Power Sector Master Plan established a comprehensive plan for the development of energy and power, including strategies for the growth of renewable energy, energy balance, and pricing (PSMP). Additionally, PSMP 2016 developed a plan and regulations for using nuclear energy, started the process of creating feed-in-tariffs, and more. Bangladesh also expects to produce up to 2470 MW of local renewable energy by 2021 and 3864 MW by 2041 under this plan. Because without the efficient use of energy, no nation can support and sustain its development [108].

## 8 Discussion

In comparison to some of the other research in this field, this work has been fairly distinctive [[26], [27], [28]]. The authors of [27,28,[109], [110], [111]] focused their discussion on Bangladesh's prospects for renewable energy resources in a very concise manner. However, no comprehensive presentation of the findings was made. The authors of [112,113] limited their discussion to Bangladesh's solar integration. Only the hydropower integration in Bangladesh was covered by the authors in Ref. [114].

However, none of them have yet disclosed the specifics of continuing renewable development, such as renewable energy plants that have already been completed and running, those that are implementation ongoing phases, and those that are still under the planning stages, and their prospects for the future. In their publications, they all provided relatively brief analyses of the current state and potential developments in renewable energy. The uniqueness of this study is that it identifies the present status of ongoing renewable energy advancement in Bangladesh in terms of RE plants that have been completed and are already operating, plants that are currently being implemented, and projects that are still in the planning stage (Table 1).

Even though the writers were able to identify challenges after reviewing the data and the accompanying documents, there were still some important problems that could not be resolved. The restriction was that the data were from official sources, but practically speaking, Bangladesh has more solar, wind, and bioenergy plants than some of the data indicate, and there were no records discovered to identify the discrepancies. In addition to this, this study does not address the challenges associated with RE installation. The old electrical power system in Bangladesh needs to be restructured in order to install these RE resources, with the creation of an electricity market that fosters competition and initiatives to guarantee energy efficiency in the context of environmental sustainability. In order to enable an effective, dependable, and responsible RE generation, transmission, and distribution system, these problems compelled electrical power corporations, governmental organizations, and the community to discover an appropriate solution.

Bangladesh, like other nations, has just recently begun to advance in renewable energy sector. The entrance levels and configurations, however, differ from those in other countries. There are still certain obstacles to developing renewable energy locally in Bangladesh, despite the existence of SREDA. The best strategy for increasing public awareness of RE is to create its popularity. As a result, holding a large number of research, training sessions, and seminars and approving the necessary funding will increase people's interest in using renewable energy rather than conventional electricity. Energy costs in the domestic sector can be reduced by educating women about the use of solar products.

Encouragement of research is crucial for achieving public procurement on RE and improving technological output, particularly when the government plans to localize renewable energies and their installation. Constrained explanations and advancements on novel technologies or procedures might shape civic perspectives, including public participation. Additionally, regional corporations, involving outside experts, design RE plants in Bangladesh. Even though such RE technology is implemented by the government and NGOs, information and public awareness are necessary to support progressive expansion for long-term aims and boost the prospects of raising in the RE sector in Bangladesh.

## 9 Conclusion

Bangladesh is experiencing an energy problem that is impeding the development of its own economy. Natural gas is used to generate around 47.91% of all electricity, but the gas supply will soon run out. The need for energy is rising, and almost 60% of its residents still suffer load shading. To meet demand in a sustainable manner, renewable energy can contribute significantly.

This review presents Bangladesh's current renewable energy situation and future prospects. According to the data presented, Bangladesh's renewable energy production is still in its infancy, but it has a promising future. In Bangladesh, renewable energy sources have the potential to produce a significant amount of electricity and close the supply-demand gap. Solar energy is practiced in varied forms in Bangladesh termed solar park, solar rooftop, solar irrigation, solar grid (minigrid, microgrid, picogrid and nanogrid), solar charging station, solar powered telecom BTS, solar home system, solar street light and solar drinking water. Strong summer winds in coastal areas can be crucial for supplying local wind energy with electricity. Already, biomass and biogas are supplying vast amounts of energy, particularly to Bangladesh's rural residents. In Bangladesh, there are roughly 307.28 million of poultry and livestock populations, where the country produces 155.82 million ton of poultry and livestock manure each year which would be the highest prospective for biogas/bioenergy generation in the country. In the hilly areas of the country, especially on the river Karnafuli, Shangu and Matamuhuri, have a lot of tributaries along with several waterfalls having suitable options for mounting of micro hydropower.

However, it is hoped that additional investments in the renewable sector will help Bangladesh meet its future energy needs. To strengthen its energy industry, Bangladesh must engage in forward-thinking planning, entice investment, and improve organizational effectiveness. So that, GoB should conscious regarding energy issues and need to reflect the visionary planning and attractive investment policy on RE in both public and private sector investment. It is noteworthy that in addition to energy production, Bangladesh also has to raise consumer knowledge and reduce electricity loss to conserve energy, which will ease the strain on the power supply.

## Author contribution statement

All authors listed have significantly contributed to the development and the writing of this article.

## Funding statement

This research did not receive any specific grant from funding agencies in the public, commercial, or not-for-profit sectors.

## Data availability statement

Data included in article/supplementary material/referenced in article.

## Declaration of interest's statement

The authors declare no conflict of interest.
