# Supplementary material for: Current status of running renewable energy in Bangladesh and future prospect: A global comparison
Source: Heliyon. 2023 Mar 15;9(3):e14308. doi: 10.1016/j.heliyon.2023.e14308 (PMC10034456; doi:10.1016/j.heliyon.2023.e14308)
Supplement: Multimedia component 1 [file mmc1.doc]

**Supplementary Figure**

Figure S1. Coal reserve of Bangladesh. [Source: Authors creation based on [20] data]

Figure S2. (a) Historical solar installed capacity from 2010 to 2020 and (b) electricity generation from 2010 to 2020 by solar energy in Bangladesh [21].

Figure S3. Present status of wind energy in Bangladesh. [Source: Authors creation based on SREDA [40] data]

Figure S4. Historical global hydropower installed capacity (GW) and new additions in 2015-2021 [19,30,82].

Figure S5. Assessing the wind speed potential of Bangladesh and measurement locations [65].
