# Supplementary material for: Current status of running renewable energy in Bangladesh and future prospect: A global comparison
Source: Heliyon. 2023 Mar 15;9(3):e14308. doi: 10.1016/j.heliyon.2023.e14308 (PMC10034456; doi:10.1016/j.heliyon.2023.e14308)
Supplement: Multimedia component 2 [file mmc2.doc]

**Supplementary Table**

Table S1. Bangladesh gas reserves [20].

| **Reserve type** | **Unit (Trillion Cubic Feet)** |
| --- | --- |
| Gas Initial in Place (GIIP) (proven + probable) | 40.09 Tcf |
| Total recoverable gas reserve (proven + probable) | 30.06 Tcf |
| Cumulative production as of December 2020 | 18.24 Tcf |
| Remaining reserve | 11.81 Tcf |

Table S2. Summary of natural gas reserve and production of Bangladesh (as of December 2020) [20].

| Gas Field | 2P GIIP  (Bcf) | 2P Reserve  (Bcf) | Gas Production in April 2020  (Bcf) | Cum. Gas  Production  (Bcf) | Remaining  Reserve  (Bcf) | Cum. Condensate  Production  (000’bbl) |
| --- | --- | --- | --- | --- | --- | --- |
| Begumganj | 47.0 | 33.0 | 0.17 | 5.6 | 27.4 | 1 |
| Shahbazpur | 415.0 | 261.0 | 0.79 | 66.0 | 195.0 | 10 |
| Semutang | 654.0 | 318.0 | 0.03 | 13.6 | 304.4 | 5 |
| Fenchuganj | 483.0 | 329.0 | 0.16 | 161.8 | 167.2 | 116 |
| Salda Nadi | 393.0 | 275.0 | 0.10 | 94.7 | 180.3 | 59 |
| Srikail* | 230.0 | 161.0 | 1.26 | 103.9 | 57.1 | 184 |
| Sundalpur* | 62.2 | 50.2 | 0.22 | 17.2 | 33.0 | 1 |
| Rupganj | 48.0 | 33.6 | 0.00 | 0.7 | 32.9 | 1 |
| Meghna | 122.0 | 101.0 | 0.22 | 75.4 | 25.6 | 121 |
| Narshingdi | 405.0 | 345.0 | 0.84 | 219.0 | 126.0 | 446 |
| Kamta | 72.0 | 50.0 | 0.00 | 21.1 | 28.9 | 4 |
| Habiganj | 3981.0 | 2,787.0 | 5.63 | 2,567.9 | 219.1 | 143 |
| Bakhrabad | 1825.0 | 1,387.0 | 1.21 | 840.2 | 546.8 | 1,046 |
| Titas | 9039.0 | 7,582.0 | 12.49 | 4,968.3 | 2,613.7 | 5,483 |
| Sangu | 976.0 | 771.0 | 0.00 | 489.5 | 281.5 | 37 |
| Bibiyana** | 8,383.0 | 4,532.0 | 41.38 | 4,506.4 | 25.6 | 27,029 |
| Moulavi Bazar** | 494.0 | 494.0 | 0.58 | 333.9 | 160.1 | 119 |
| Jalalabad** | 2,716.0 | 2,716.0 | 6.52 | 1,428.3 | 1,287.7 | 10,825 |
| Feni | 185.0 | 130.0 | 0.00 | 63.0 | 67.0 | 110 |
| Kailas Tila | 3,463.0 | 2,880.0 | 1.30 | 766.4 | 2,113.6 | 8,143 |
| Sylhet | 580.0 | 408.0 | 0.12 | 217.0 | 191.0 | 814 |
| Rashidpur | 3,887.0 | 3,134.0 | 1.25 | 662.4 | 2,471.6 | 807 |
| Chattak | 677.0 | 474.0 | 0.00 | 25.8 | 448.2 | 4 |
| Beani Bazar | 225.0 | 137.0 | 0.25 | 101.3 | 35.7 | 1,664 |
| Bangura | 730.0 | 621.0 | 2.80 | 493.1 | 127.9 | 1265 |
| Kutubdia | 65.0 | 46.0 | 0.00 | 0.0 | 46.0 | 0 |
| **Total** | **40,092.2** | **30,055.4** | **77.32** | **18,242.8** | **11,812.6** | **58,436** |

** Preliminary reserve estimated by BAPEX.*

*** 2P reserve estimation by Petrobangla has crossed the limit. So 2P reserve is estimated as GIIP reserve now.*

Table S3. Average wind speed in different places of Bangladesh. [Authors creation based on BMD [63] purchased data]

| Average speed (kph) at 50m height | | | | | | | | | | | | | | | | | | | |
| --- | --- | --- | --- | --- | --- | --- | --- | --- | --- | --- | --- | --- | --- | --- | --- | --- | --- | --- | --- |
|  | **Tekhnaf** | **Sitakunda** | **Sandwip** | **M. Court** | **Kutubdia** | **Chittagong** | **Hatiya** | **Feni** | **Cox's Bazar** | **Chandpur** | **Patuakhali** | **Khepupara** | **Bhola** | **Barisal** | **Mongla** | **Khulna** | **Chuadanga** | **Jessore** | **Satkhira** |
| **Jan** | 4.8 | 4.13 | 4.35 | 3.77 | 3.77 | 4.55 | 4.2 | 3.59 | 5.48 | 3.9 | 4.26 | 4.31 | 4.09 | 4.17 | 3.45 | 4.03 | 4.09 | 4.49 | 4.16 |
| **Feb** | 5.49 | 4.1 | 4.95 | 4.16 | 3.92 | 4.54 | 4.65 | 3.86 | 4.98 | 4.54 | 4.21 | 4.34 | 4.26 | 4.31 | 3.9 | 4.13 | 4.26 | 4.88 | 4.26 |
| **Mar** | 6.62 | 4.77 | 5.85 | 5.14 | 4.58 | 5.19 | 4.95 | 5.22 | 4.62 | 4.72 | 5.48 | 5.65 | 5.08 | 5.11 | 5.1 | 4.85 | 4.75 | 5.44 | 5.08 |
| **Apr** | 6.66 | 4.89 | 7.05 | 5.52 | 5.18 | 6.07 | 6.15 | 6.5 | 4.76 | 7.15 | 6.14 | 6.19 | 6.22 | 6.37 | 6.6 | 6.04 | 5.47 | 6.24 | 6.22 |
| **May** | 5.99 | 5.25 | 6.9 | 5.91 | 5.49 | 5.86 | 6.6 | 6.12 | 5.48 | 5.89 | 6.71 | 6.94 | 6.71 | 6.52 | 6.3 | 6.19 | 6.22 | 6.37 | 6.45 |
| **Jun** | 5.55 | 5.61 | 8.55 | 6.42 | 6.41 | 7.97 | 7 | 6.34 | 7.17 | 5.26 | 7.42 | 7.79 | 7.37 | 7.4 | 5.85 | 6.53 | 6.71 | 7.24 | 6.71 |
| **Jul** | 4.8 | 5.78 | 8.85 | 6.32 | 6.42 | 8.06 | 7.05 | 6.02 | 7.4 | 4.39 | 7.6 | 8.15 | 7.61 | 7.61 | 5.25 | 6.45 | 6.43 | 6.79 | 6.39 |
| **Aug** | 4.65 | 5.04 | 7.8 | 5.65 | 5.64 | 7.11 | 5.85 | 5.44 | 6.66 | 4.24 | 6.71 | 7.55 | 6.66 | 6.88 | 5.1 | 5.96 | 5.78 | 6.3 | 5.57 |
| **Sep** | 4.04 | 4.08 | 5.85 | 4.81 | 4.32 | 5.34 | 4.95 | 4.81 | 5.21 | 4.29 | 5.89 | 6.27 | 5.08 | 5.12 | 4.2 | 4.39 | 4.75 | 4.88 | 4.42 |
| **Oct** | 3.62 | 3.89 | 4.8 | 3.98 | 3.45 | 4.32 | 3.75 | 3.26 | 4.62 | 3.11 | 4.32 | 4.44 | 3.93 | 3.88 | 3.45 | 3.95 | 4.75 | 4.13 | 3.6 |
| **Nov** | 3.92 | 3.38 | 4.5 | 3.67 | 3.06 | 4.17 | 3 | 3.14 | 4.98 | 3.6 | 4.06 | 4.11 | 3.6 | 3.67 | 3.6 | 3.54 | 3.65 | 3.86 | 3.93 |
| **Dec** | 4.35 | 3.53 | 4.35 | 3.32 | 3.23 | 4.24 | 3.18 | 3.29 | 5.27 | 4.99 | 4.13 | 4.17 | 3.93 | 3.9 | 4.05 | 3.98 | 4.09 | 4.14 | 4.09 |
| **AVE** | **5.04** | **4.54** | **6.15** | **4.89** | **4.62** | **5.62** | **5.11** | **4.79** | **5.55** | **4.67** | **5.57** | **5.82** | **5.38** | **5.41** | **4.74** | **5.00** | **5.07** | **5.39** | **5.07** |

Table S4. Global production of biofuels, top 15 countries and EU-28, 2019 [19].

| Country | Ethanol | Biodiesel (FAME) | Biodiesel (HVO) | Change relative to 2018 |
| --- | --- | --- | --- | --- |
|  | Billion litres | | | |
| United States | 59.7 | 4.0 | 2.5 | -1.7 |
| Brazil | 35.3 | 5.9 | 0.0 | 2.9 |
| Indonesia | 0.0 | 7.9 | 0.0 | 3.9 |
| China | 4.0 | 0.6 | 0.0 | 0.7 |
| Germany | 0.8 | 3.8 | 0.0 | 0.0 |
| France | 0.9 | 2.8 | 0.2 | -0.3 |
| Argentina | 1.1 | 2.5 | 0.0 | -0.2 |
| Thailand | 1.6 | 1.7 | 0.0 | 0.3 |
| Spain | 0.5 | 2.0 | 0.0 | 0.1 |
| Netherlands | 0.4 | 1.0 | 1.1 | 0.1 |
| Canada | 2.0 | 0.3 | 0.0 | 0.3 |
| India | 2.1 | 0.2 | 0.0 | 0.5 |
| Malaysia | 0.0 | 1.6 | 0.0 | 0.7 |
| Poland | 0.2 | 1.0 | 0.0 | 0.1 |
| Italy | 0.0 | 0.8 | 0.2 | 0.2 |
| EU-28 | 4.7 | 12.4 | 2.9 | -0.1 |
| **World Total (2019)** | **113.3** | **48.5** | **6.9** | **7.5** |
| World Total (2018) | 111.9 | 38 | 7.3 | 9.9 |
